# Prevalence of lower back pain and its associations with lifestyle behaviors among college students in Saudi Arabia

**DOI:** 10.1186/s12891-023-06683-5

**Published:** 2023-08-11

**Authors:** Mohammed M Alshehri, Amjad M Alqhtani, Shahd H Gharawi, Raghad A Sharahily, Wajd A Fathi, Shahad G Alnamy, Shaima A Alothman, Yasir S. Alshehri, Ahmed S. Alhowimel, Bader A. Alqahtani, Aqeel M. Alenazi

**Affiliations:** 1 Physical Therapy Department, Jazan University, Jazan, 82412 Saudi Arabia; 2 Medical Research Center, Jazan University, Jazan, 45142 Saudi Arabia; 3 Lifestyle and Health Research Center, Health Science Research Center, Princess Nourah bint Abdulrahman University, Riyadh, Saudi Arabia; 4 Department of Physical Therapy, College of Medical Rehabilitation Sciences, Taibah University, Madinah, Saudi Arabia; 5 Department of Health and Rehabilitation Sciences, Prince Sattam Bin Abdulaziz University, Alkharj, Saudi Arabia

**Keywords:** Lower back pain, Lifestyle, College, Quality of life

## Abstract

**Background:**

Lower back pain (LBP) is a common musculoskeletal disorder that may affect students’ daily lives. Recent psychological research showed a relevant connection between LBP and multidimensional health. However, the association between LBP and lifestyle behavior has not been established, and improving knowledge in this area may help develop preventive strategies and optimize college students’ quality of life.

**Methods:**

A cross-sectional study of 1420 college students in Saudi Arabia was conducted, and participants who attended Saudi Universities were recruited from May 2021 to November 2021. An established validated online survey assessed LBP, sleep quality, time spent sedentary (sedentary duration), health responsibility, physical activity, nutrition, spiritual growth, interpersonal relationships, and stress management. Generalized Linear Regression was used to assess the associations between LBP severity and lifestyle behaviors after controlling for covariates.

**Results:**

LBP was prevalent among college students from Saudi Arabia. Most of the sample were young (23.81 ± 6.02), and female (83.7%). There were significant differences between students with and without LBP regarding age, BMI, sex, marital status, pain severity, overall lifestyle behavior, health responsibility, physical activity, nutrition, stress management, and global sleep quality. After controlling for age, BMI, sex, and marital status, there were significant associations between pain severity and global sleep quality (ß=0.2, p < .001, CI: 16 to 0.24), and sedentary duration (ß=0.03, p = .01, CI:0.009 to 0.06).

**Conclusions:**

This study helped define the prevalence of LBP in college students in Saudi Arabia and evaluated the association between LBP and lifestyle behaviors. The findings showed that students with higher levels of poor sleep quality or sedentary behavior had higher levels of pain. Promoting sleep quality and reducing sedentary behavior may help establish preventive strategies for LBP in college students.

## Background

Low back pain (LBP) is a multifactorial disorder related to individual, physical and psychosocial work, and environmental factors [1]. Worldwide estimates of the lifetime prevalence of LBP vary from 60 to 80% [2]. Some studies reported a high point prevalence (i.e., at the time of the study) of LBP among university students, which could reach up to 41.2% [3–8]. Many university students may experience LBP due to prolonged sitting while studying or working on computers [3, 4]. Several factors may explain these associations, including obesity, income, sedentary lifestyle, physical inactivity, and poor self-care [9–12]. LBP is associated with multidimensional lifestyle issues which have not been investigated among university students. Since up to 80% of the general population may experience LBP at some point in their lives [13], university students, too, are subjected to LBP.

Few studies have investigated LBP incidence and prognosis in university students. A study in nursing students has shown that physical activity level, slump sitting, stress were among the predictors of LBP [14]. Another study among medical students (n = 160) found that LBP was associated with poor physical activity and psychological factors [15]. While another study on medical students (n = 629) found that LBP was associated with psychological factors not physical activity [16]. Overall, most of these studies did not assess the whole spectrum of lifestyle behaviors. Further, these studies focused mainly on medical students where it is expected that they have a higher health literacy compared to other university students.

Physical activity and LBP, directly and indirectly, affect young adults [17]. Physical activity is highly associated with LBP. It is one of the most important health elements for people with LBP, which may affect their physical activity in daily life [17]. Students are more likely to engage in unhealthy behaviors that adversely affect their well-being, such as physical inactivity, stress, and sedentary behavior [18]. According to Keating’s research, physical inactivity affects 40-50% of college students [19]. According to a recent survey, only 9% of Czech university students met the 10,000 step-per-day requirement [20].

A sedentary lifestyle significantly increased the incidence of recurrent LBP, while increased physical activity significantly affected the occurrence of chronic LBP [21]. Another study conducted in the United Kingdom found that university students spent eight hours per day on sedentary activities such as learning, watching television, gaming, computer activities, and sitting [22]. Knowledge is scarce on how to reduce sedentary behavior among university students [23]. Prolonged sitting (i.e., sedentary behavior) may be associated with overload and stress in students during their life [24]. One study in Saudi Arabia reported that 61.5% of students who sit for prolonged periods (over 4 h per day) have commonly reported LBP [25]. However, there is limited evidence of the relationship between potential lifestyle factors and LBP.

Sleep loss was linked to an unhealthy lifestyle (i.e., lack of exercise, poor health, and irregular eating habits) [26]. Almost half of university students (48.7%) in Poland reported sleeping from 5 to 7 h [27]. Sleep problems and pain intensity are intimately linked; 50–60% of patients with sleep problems reported LBP [28]. It has also been reported that persons with LBP with poor sleep quality and more severe pain are at a higher risk of being hospitalized for care than those with good sleep quality [29]. These findings suggest that poor sleep quality may be associated with the development of LBP. The estimated prevalence of sleep disturbance was 58.7% in patients with LBP [30]. Sleep disturbance was found to be independently associated with pain intensity, where each increase by one point on a ten-point pain scale was associated with a 10% increase in the likelihood of reporting poor sleep quality [30].

The biopsychosocial model of health has been shown to be predictive of LBP incidence and prognosis [31]. However, it is does not fully explain the role of lifestyle behaviors combined in LBP incidence and prognosis [32]. Thus, investigating lifestyle behaviors among students with LBP is imperative because of the associated health complications with LBP for young adults. Since students involve in physical and psychological changes during their university life, a healthy lifestyle behavior may contribute as an important factor in minimizing these changes and staying physically and psychologically stable. Understanding the complex relationship between LBP and lifestyle behaviors may help future research to establish preventive strategies and treatment plans for university students with LBP. Therefore, we aimed to determine the prevalence of LBP and the associations between LBP and lifestyle outcomes in university students.

## Methods

### Design

**Fig. 1:**
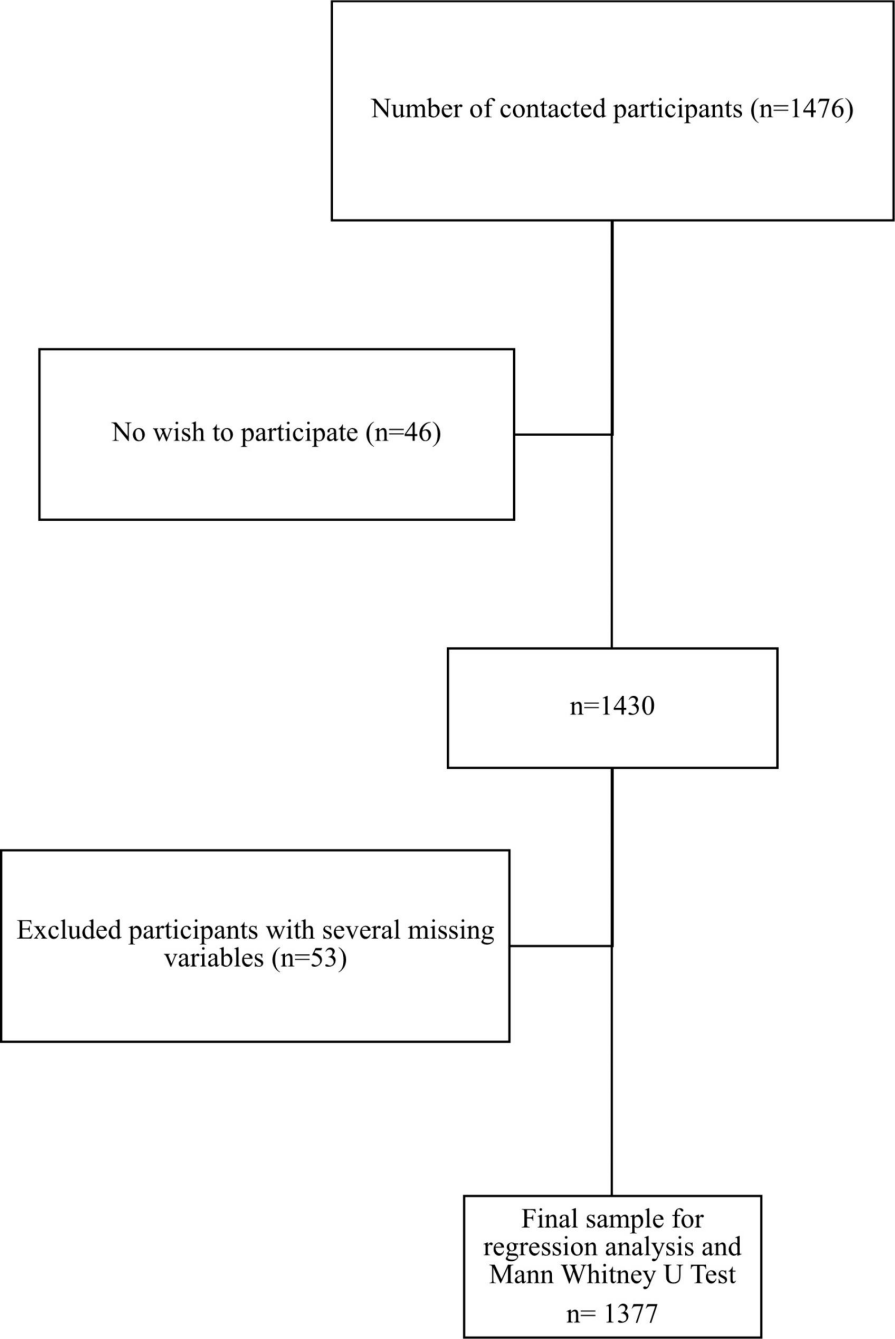
Flowchart of the study

This was a cross-sectional study of 1420 college students attending Saudi universities. Figure 1 shows the flowchart of the study which illustrated the number of students enrolled and finally analyzed. Random selection criteria were used to recruit students from different sites. Online surveys were accepted by 1420 of the 1476 individuals, giving a response rate of 96.88% (i.e., response rate = no accept to participate/accepted participants). We used different approaches such as emails and social media (e.g., Twitter, personalized WhatsApp messages) to recruit students. The study was approved by the Research Ethics Committee of the Physical Therapy Department at Jazan University (No. PHT05001S017). Questionnaires were created and designed through the Google Forms website to reach the highest number of students between May and November 2021. On the first page of the survey, we included the study’s purpose, eligibility, and description. Interested participants were provided with an informed consent statement at the end of the study description and were required to select “yes” to access the questions of the survey. The survey was composed of different sections that asked about the demographics of students, the presence of low back pain, pain scale, the 19-item Pittsburgh Sleep Quality Index, the 52-item Health-Promoting Lifestyle Profile-II, and a single question about sedentary duration.

### Participants

All college students in the Kingdom of Saudi Arabia were asked to fill out the online survey to capture real-world validity. Random samples were taken from each site (i.e., the number of students for each university) and checked regularly to ensure equal site contributions to this study. We included college students who were 18 years or older and active in person students. We excluded students who underwent surgical treatment for physical complaints other than back pain, had a BMI > 45, and/or were pregnant. All participants provided informed consent prior to starting the survey.

### Outcomes

- Demographics: Sociodemographic characteristics (age, sex, university name, weight, height, and marital status).
- Back pain: a yes/no question was asked to determine the presence of low back pain.
- Pain severity: A scale of zero to 10 was used to identify the severity of pain symptoms for low back pain.
- Sleep: Sleep quality was assessed using the Pittsburgh Sleep Quality Index (PSQI), which has been tested for validity and reliability [33]. The PSQI assesses overall sleep quality over the previous month and consists of 19 individual items, creating seven components that produce one global score ranging from 0 to 21 [33]. Poor sleepers have scores ≥ 5 as a global cutoff score.
- Lifestyle behaviors: Health Promoting Lifestyle Profile II (HPLP-II) was utilized to identify the lifestyle behavior [34]. HPLP-II has six sub-scales (health responsibility, physical activity, nutrition, spiritual growth, interpersonal relationships, and stress management) using a 4-point Likert scale with 52 items. A higher score on HPLP-II indicated a higher level of health-promoting behavior. It has been recommended to average six sub-scales to calculate overall lifestyle behavior [34].
- Sedentary duration: A question was designed to ask participants the amount of time in hours (hrs) per day spent sitting without being active in the past week.

### Statistical analysis

All the data in this study was transferred from the Google Forms into Microsoft Excel 2019. Continuous variables are conveyed as mean ± SD, and categorical variables are presented as the frequency for the whole sample using SPSS version 25. All data was scattered based on the presence of LBP (group) to assess the differences in demographics and lifestyle behaviors between students with and without LBP. Normality of data was tested in which anormal distributed data analyses were appropriate for this study. Non-normal data for unequal samples were evaluated using the Mann-Whitney U test, and frequencies and percentages were assessed using the Chi-squared test. The Linear Generalized Regression Model was used to determine the association between LBP severity and lifestyle behaviors after controlling for covariates: Model 1 (no covariates), Model 2 (age), and Model 3 (Age, BMI, sex, and marital status) with a significance level of P < .05.

## Results

In Table 1, most of the sample are young (23.81 ± 6.02), within a healthy weight category (23.29 ± 5.48), female (83.7%), and single (72%). The data revealed a high parentage of self-reported LBP (66.4%) with moderate-to-average pain severity for the whole sample (4.03 ± 2.66). Most students had poor sleep quality (7.33 ± 3.60) and were sedentary (8.58 ± 4.77).

**Table 1:** Demographics and clinical characteristics for whole sample size

Variables	Mean ± SD; N (%)
Age (N = 1377)	23.81 ± 6.02
BMI (N = 1360)	23.29 ± 5.48
Sex, Female	1155 (83.7)
Marital Status	
Single Married Divorced Widow	994 (72) 352 (25.5) 28 (2.0) 6 (0.4)
Back pain, yes (N = 1380)	916 (66.4)
Pain severity (N = 1380)	4.03 ± 2.66
HPLP total (N = 1380)	22.52 ± 4.64
Health Responsibility (N = 1380)	21.19 ± 6.30
Physical Activity (N = 1380)	17.15 ± 5.92
Nutrition (N = 1380)	21.60 ± 5.50
Spiritual growth (N = 1380)	27.58 ± 5.82
Interpersonal relations (N = 1380)	26.54 ± 5.15
Stress management (N = 1380)	21.05 ± 5.07
Sleep duration (N = 1360)	7.21 ± 2.10
Global sleep quality (N = 1300)	7.33 ± 3.60
Sedentary duration, hrs (N = 1360)	8.58 ± 4.77

In the comparison between students with and without low back pain (LBP), significant differences were found for age, BMI, sex, marital status, pain severity, HPLP-II total score, HPLP-II subscales (health responsibility, physical activity, nutrition, stress management), and global sleep quality. Students with LBP were older (24.24 ± 6.47) compared to those without LBP (22.96 ± 4.90), had a higher BMI (23.60 ± 5.77) compared to (22.69 ± 4.80), and a greater severity of LBP (4.98 ± 2.34) compared to no LBP (2.16 ± 2.22). In addition, students with LBP had lower scores for lifestyle behaviors, including health responsibility, physical activity, nutrition, stress management and global sleep quality, compared with students without LBP. Furthermore, female sex was identified to be more prevalent among students with LBP (86.74%), compared to those without LBP (78.23%), and this difference was statistically significant (P < .001) (Table 2).

**Table 2:** The differences between students with and without back pain

Variables	Low Back Pain (N = 913)	No Low Back Pain (N = 464)	P-value
Age	24.24 ± 6.47	22.96 ± 4.90	0.01
BMI	23.60 ± 5.77	22.69 ± 4.80	0.02
Sex, Female	792 (86.74)	363 (78.23)	< 0.001
Marital Status			< 0.001
Single Married Divorced Widow	626 (68.56) 269 (29.46) 17 (1.86) 4 (0.44)	368 (79.31) 83 (17.88) 11 (2.37) 2 (0.43)	
Pain severity	4.98 ± 2.34	2.16 ± 2.22	< 0.001
HPLP-II total score	22.25 ± 4.63	23.03 ± 4.61	0.003
Health Responsibility	20.94 ± 6.26	21.68 ± 6.36	0.03
Physical Activity	16.77 ± 5.76	17.90 ± 5.76	0.002
Nutrition	21.30 ± 5.42	22.18 ± 5.62	0.006
Spiritual growth	27.38 ± 5.96	27.96 ± 5.49	0.2
Interpersonal relations	26.40 ± 5.20	26.80 ± 5.05	0.27
Stress management	20.74 ± 5.19	21.65 ± 4.78	0.001
Sleep duration, hrs	7.20 ± 2.09	7.23 ± 2.07	0.67
Global sleep quality	7.84 ± 3.64	6.33 ± 3.27	< 0.001
Sedentary duration, hrs	8.69 ± 5.00	8.35 ± 4.27	0.11

Table 3 presents the results of the multiple linear regression models. Across all three models, global sleep quality was consistently significant, with a ß value of 0.22 in model 1 and 0.21 in model 2 and model 3. In model 1, physical activity had a significant negative relationship with low back pain (ß=-0.05, p = .005), indicating that students with higher levels of physical activity had lower levels of pain. However, this relationship was not significant in models 2 and 3. Sedentary duration was also significantly associated with low back pain in model 1 (ß=0.03, p = .03) and model 2 (ß=0.04, p = .006) but was not significant in model 3. None of the other variables had significant associations with low back pain.

**Table 3:** The associations between pain severity and lifestyle behaviors

Predictors	ß	SE	CI (95%)	P value
Model 1				
Health Responsibility Physical Activity Nutrition Spiritual growth Interpersonal relations Stress management Global sleep quality Sedentary duration, hrs	0.01 − 0.05 − 0.001 0.03 0.03 0.03 0.22 0.03	0.02 0.01 0.02 0.02 0.02 0.02 0.02 0.01	− 0.02 to 0.05 − 0.09 to 0.01 − 0.04 to − 0.04 − 0.07 to 0.01 − 0.02 to 0.07 − 0.04 to 0.06 0.18 to 0.26 0.003 to 0.06	0.43 0.005 0.97 0.15 0.22 0.65 < 0.001 0.03
Model 2				
Health Responsibility Physical Activity Nutrition Spiritual growth Interpersonal relations Stress management Global sleep quality Sedentary duration, hrs	0.008 − 0.05 − 0.01 − 0.03 0.02 0.02 0.21 0.04	0.01 0.01 0.02 0.02 0.02 0.02 0.02 0.01	− 0.03 to 0.04 − 0.08 to − 0.009 − 0.05 to 0.03 − 0.07 to 0.01 − 0.02 to 0.06 − 0.03 to 0.06 0.17 to 0.25	0.67 0.01 0.63 0.16 0.24 0.54 < 0.001 0.006
Model 3				
Health Responsibility Physical Activity Nutrition Spiritual growth Interpersonal relations Stress management Global sleep quality Sedentary duration, hrs	0.006 − 0.03 − 0.02 − 0.03 0.02 0.01 0.2 0.03	0.01 0.02 0.02 0.02 0.02 0.02 0.02 0.01	− 0.03 to 0.04 − 0.07 to 0.004 − 0.06 to 0.02 − 0.07 to 0.01 − 0.02 to 0.06 − 0.04 to 0.06 0.16 to 0.24 0.009 to 0.06	0.75 0.08 0.27 0.14 0.28 0.71 < 0.001 0.01

Dependent variable: pain severity

Model 1: no covariates

Model 2: Age

Model 3: Age, BMI, sex, and marital status

## Discussion

The main objectives of this cross-sectional study were to estimate the prevalence of LBP and examine the relationship between lifestyle outcomes and LBP among students in Saudi universities. The current study showed a high prevalence of LBP among students (66%). In addition, most of the current sample had poor sleep quality and a sedentary lifestyle (7.3%, and 8.6%, respectively). The prevalence of LBP was higher in female students, students with poor sleep quality, and students with poor stress management or lower physical activity.

LBP is thought to be triggered by factors such as the repetitive strain of a specific body area and extended standing or sitting postures. However, the most significant elements that contribute to the development of LBP and its progression to chronicity are as follows: various stressors, a fear of pain, and a lack of physical activity [35, 36]. Therefore, students are at greater risk of developing LBP due to the academic environment requiring extended hours per day attending lectures and studying in an uninterrupted sitting position [37].

Our research revealed that LBP is prevalent among students across Saudi Arabian universities. Similar percentages have been seen in other countries; 32.5% of undergraduate students at a medical college in Delhi and 34.6% of medical students studying at a university in Turkey had LBP [37]. Moreover, the prevalence of LBP among medical students in Saudi Arabia ranged from 40.5% in university hospitals in Riyadh to 52.5% at Jazan University [38, 39]. In contrast to these findings, a Malaysian study showed the prevalence of LBP among Malaysian medical students to be 27.2% [40]. LBP was consistently found to be 17.9% among fourth-year medical students in China [41] and 17.2% at Belgrade University in Serbia [42]. LBP was less common among Brazilian medical students, with a frequency of 9.2% [43]. Other studies discovered a 13% incidence rate among medical students at a Pakistani college [44]. It should be mentioned that most research on the prevalence of LBP among university students has been undertaken among medical students; nevertheless, our study highlights the prevalence across all university students. Demographics, study methodology, academic curriculum, as well as cultural, educational, lifestyle, and dietary factors, may all have a role in the occurrence of LBP across studies.

Unhealthy lifestyle behavior can have a negative impact on LBP. A cross-sectional United States population study indicated that moderate physical activity and sedentarism were associated with this discomfort. Another cross-sectional study conducted in the U.S revealed that physical activity (even at low levels), cigarette usage, alcohol consumption, sleep quality, and BMI are all connected to LBP [45, 46]. Similarly, national-level studies on LBP in Iran, Canada, and Spain show the influence of lifestyle behavior [47–49]. Longitudinal studies, on the other hand, have revealed weak or insignificant associations.

Understanding the role of healthy lifestyle behaviors in the risk of LBP could have substantial public health implications, as it could provide information on public health preventative activities. For instance, in a Swedish cohort study on (n = 12483) healthy lifestyle behaviors in a group free of LBP showed that a healthy lifestyle may protect against long-term bothersome back or neck pain four years later [50]. In another cohort study of more than 8000 men and women with a follow-up of four years, healthy lifestyle behaviors improved the prognosis of women with occasional LBP. Women with one healthy lifestyle component had a 35% lower chance of developing long-term troublesome LBP, while women with all four healthy lifestyle components had a 52% lower risk of developing long-term LBP. Compared to women with unhealthy lifestyle behavior, the proportion of women with LBP was 5% lower at follow-up if they had one healthy lifestyle factor and 8% lower if they had four healthy lifestyle factors [51]. Thus, it is sensible to promote a healthy lifestyle among university students, given that the nature of the educational environment cannot be changed.

Our results indicated that students with no LBP reported being more health responsible and chose to eat healthily. Further, no significant associations with pain severity were found. The multifactorial nature of LBP may explain the observed results. The incidence and prognosis of LBP are greatly affected by a cluster of physical, psychological, social, and health-related quality of life factors [52, 53]. Thus, being health reasonable, adopting a healthy lifestyle is to act in one’s own interest [54] and may decrease the incidence of developing LBP. However, health responsibility is not enough to influence pain severity.

Previous research has linked LBP to poor sleep quality or sleep disorders [55–58]. These findings were consistent with our study that found that people with LBP have worse sleep quality. Recent work has found that the prevalence of poor sleep quality was higher in those with moderate and severe LBP and in male soldiers in Saudi Arabia. Although previous work has found a bidirectional relationship between sleep quality and pain severity [56], our study could not examine this association. The mean difference in our study was 1.5 in the global score for PSQI. However, this mean difference is lower than the minimal clinically important differences for PSQI, ranging from 3.10 to 4.4 in different populations [59, 60]. Our findings indicate that each one-point increase in PSQI was associated with a 0.20 increase in back pain severity, but this increase is not clinically meaningful. Clinically, past evidence has shown that improved sleep quality over time was associated with improved LBP severity [61]. Therefore, targeting pain severity and/or sleep quality might result in greater improvements. Future research should examine the bidirectional relationship of factors affecting improvements in pain severity and sleep quality.

Our findings showed a significant difference in physical activity between students with LBP and those without. These findings were consistent with prior studies that found an association between LBP and a lack of physical activity [62–64]. However, other studies have reported no association between physical activity and LBP [65–67]. In addition, after controlling for a possible confounder, our results showed no association between physical activity and LBP. These contradictory findings can be explained by the fact the relationship between physical activity and LBP may be U shaped [68]. Both sedentary lifestyle and high-intensity physical activities raised the incidence of LBP. In addition, the different methodologies used to quantify physical activity levels can explain such discrepancies in the findings. Using an objective measure to assess physical activity level can yield more accurate and reliable estimates than a self-reported question/answer [69]. Physical inactivity is frequently a risk factor, particularly for developing LBP disorders. Therefore, a longitudinal study using an objective tool to assess the association between physical activity levels and LBP is warranted.

Our results indicate that university students spend more than eight hours daily engaging in sedentary behavior. Similar to our findings, a recent systematic review showed that the average university student reported engaging in sedentary behavior for about seven hours per day. Time spent sedentary increased to > 9 h when measured objectively [70]. However, our results indicated no difference between people with LBP and those without in average sedentary time. The association between sedentary behavior and LBP is still debated in the literature. A study on 2,148 twins [71] and another with 330 adolescents [72] showed no difference between sedentary behavior and LBP while a study with 479 office workers indicated sitting for more than ten hours per day is associated with LBP [73]. This debate in the literature might be explained by our results that indicate sedentary behavior is positively associated with pain severity. Few interventional studies showed initial support for less sitting and its linked to LBP pain severity [74, 75]. Future studies may be required to establish if being sedentary is a risk factor for developing LBP or if it can lead to a worse LBP prognosis.

The main strength of this study was that the survey was distributed to students at several universities around the country and was not limited to students at one institution. On the other hand, our study has several limitations. First, the cross-sectional study design cannot infer a causal relationship between risk factors and LBP. Moreover, sampling in cross-sectional studies might not accurately reflect a wider population. Therefore, there is a need to develop a longitudinal study. Second, although the study utilized valid and reliable outcome measures for sleep and lifestyle, the data collection was still based on self-reported information, and, therefore, the risk of bias may be present. Third, the students were asked about their experience with LBP at the time of answering the survey. Therefore, future studies should include questions about the lifetime prevalence and last 12-month prevalence of LBP and their associations with students’ lifestyle outcomes. Fourth, we also did not collect data about the university year level or educational programs that the students were enrolled in. Investigating the associations between the incidence of LBP and university year level (e.g., first-year students vs. final-year students) and educational programs (e.g., classroom-based vs. classroom- and practical/clinic-based courses) would provide additional information about which group of students is at risk of LBP. Finally, it might be imperative to identify the associations of regional pain mapping and the lifestyle behaviors, which will help clinicians identifying other risk factors.

## Conclusions

The prevalence of LBP was relatively high (66.4%), with moderate pain severity among college students. This study found that students with LBP were older, had a higher BMI, higher pain severity, and lower positive lifestyle behavior when compared to those without LBP. This study showed associations between increased LBP severity, decreased physical activity level, poor sleep quality, and high sedentary duration. Further research is needed to examine the bidirectional relationship between pain severity, activity level, and sleep parameters using objective measures.

## Data Availability

The datasets generated and/or analyzed during the current study are not publicly available due the confidentiality of the participants’ data but are available from the corresponding author on reasonable request.

## Abbreviations

HPLP II: Health Promoting Lifestyle Profile II
LBP: Lower Back Pain
PSQI: Pittsburgh Sleep Quality Index

## References

[1] Kirsch Micheletti J (2019). Association between lifestyle and musculoskeletal pain: cross-sectional study among 10,000 adults from the general working population. BMC Musculoskelet Disord.

[2] Lee P (2001). Low back pain: prevalence and risk factors in an industrial setting. J Rhuematol.

[3] Daldoul C (2020). AB0962 Low back pain among medical students: prevalence and risk factors. Ann Rheum Dis.

[4] AlShayhan FA, Saadeddin M (2018). Prevalence of low back pain among health sciences students. Eur J Orthop Surg Traumatol.

[5] Vujcic I et al. *Low Back Pain among Medical Students in Belgrade (Serbia): A Cross-Sectional Study* Pain Res Manag, 2018. 2018: p. 8317906. 10.1155/2018/8317906 PMC5829428 29623146

[6] Aggarwal N (2013). Low back pain and associated risk factors among undergraduate students of a medical college in Delhi. Educ Health (Abingdon).

[7] Falavigna A (2011). Increased prevalence of low back pain among physiotherapy students compared to medical students. Eur Spine J.

[8] Hafeez K (2013). Back Pain - Are Health Care Undergraduates at risk?. Iran J Public Health.

[9] Hoy D (2010). The epidemiology of low back pain. Best Pract Res Clin Rheumatol.

[10] Dighriri YH (2019). Prevalence and associated factors of neck, shoulder, and low-back pains among medical students at Jazan University, Saudi Arabia: a cross-sectional study. J Family Med Prim Care.

[11] Burton AK (1995). Psychosocial predictors of outcome in acute and subchronic low back trouble. Spine (Phila Pa 1976).

[12] Wong AYL (2021). Prevalence/Incidence of low back Pain and Associated Risk factors among nursing and medical students: a systematic review and Meta-analysis. Pm r.

[13] Rubin DI (2007). Epidemiology and risk factors for spine pain. Neurol Clin.

[14] Mitchell T (2010). Identification of modifiable personal factors that predict new-onset low back pain: a prospective study of female nursing students. Clin J Pain.

[15] Aggarwal N (2013). Low back pain and associated risk factors among undergraduate students of a medical college in Delhi. Educ health.

[16] Tavares C (2019). Low back pain in brazilian medical students: a cross-sectional study in 629 individuals. Clin Rheumatol.

[17] Verbunt JA (2001). Physical activity in daily life in patients with chronic low back pain. Arch Phys Med Rehabil.

[18] Almutairi KM (2018). Health promoting lifestyle of university students in Saudi Arabia: a cross-sectional assessment. BMC Public Health.

[19] Keating XD (2005). A meta-analysis of college students’ physical activity behaviors. J Am Coll Health.

[20] Sigmundová D (2013). Physical activity in the lifestyle of czech university students: meeting health recommendations. Eur J Sport Sci.

[21] Citko A et al. *Sedentary Lifestyle and Nonspecific Low Back Pain in Medical Personnel in North-East Poland* Biomed Res Int, 2018. 2018: p. 1965807. 10.1155/2018/1965807 PMC6151221 30271778

[22] Deliens T (2015). Determinants of physical activity and sedentary behaviour in university students: a qualitative study using focus group discussions. BMC Public Health.

[23] Franco DC, Ferraz NL, Sousa TFd. Sedentary behavior among university students: a systematic review. Revista Brasileira de Cineantropometria & Desempenho Humano; 2019. p. 21.

[24] Beach TA (2005). Effects of prolonged sitting on the passive flexion stiffness of the in vivo lumbar spine. Spine J.

[25] Felemban RA (2021). Prevalence and predictors of Musculoskeletal Pain among undergraduate students at a Dental School in Saudi Arabia. Clin Cosmet Invest Dentistry.

[26] Ohida T (2001). The influence of Lifestyle and Health Status factors on sleep loss among the Japanese General Population. Sleep.

[27] Jakubiec D, et al. Lifestyle of students from different universities in Wroclaw, Poland. Volume 66. Roczniki Państwowego Zakładu Higieny; 2015. 4. 26656415

[28] Takahashi M, Matsudaira K, Shimazu A (2015). Disabling low back pain associated with night shift duration: sleep problems as a potentiator. Am J Ind Med.

[29] Jolfaei AG, Makvandi A, Pazouki A (2014). Quality of sleep for hospitalized patients in Rasoul-Akram hospital. Med J Islamic Repub Iran.

[30] Alsaadi SM (2011). Prevalence of sleep disturbance in patients with low back pain. Eur Spine J.

[31] Otero-Ketterer E (2022). Biopsychosocial factors for chronicity in individuals with non-specific low back Pain: an Umbrella Review. Int J Environ Res Public Health.

[32] Van Tulder MKM (2007). Low back pain (nonspecific). Best Pract Res Clin Rheumatol.

[33] Suleiman KH (2010). Translating the Pittsburgh sleep quality index into Arabic. West J Nurs Res.

[34] Walker SN, Sechrist KR, Pender NJ. The health-promoting lifestyle profile: development and psychometric characteristics. Nursing research; 1987. 3644262

[35] Gallagher S, Heberger JR (2013). Examining the interaction of force and repetition on musculoskeletal disorder risk: a systematic literature review. Hum Factors.

[36] Shrier I (2001). Risk factors for development of lower limb pain in adolescents. J Rhuematol.

[37] Hosteng KR (2019). Uninterrupted classroom sitting is associated with increased discomfort and sleepiness among college students. Int J Environ Res Public Health.

[38] Algarni AD et al. *The prevalence of and factors associated with neck, shoulder, and low-back pains among medical students at university hospitals in Central Saudi Arabia* Pain Research and Treatment, 2017. 2017. 10.1155/2017/1235706 PMC5697379 29238618

[39] Dighriri YH (2019). Prevalence and associated factors of neck, shoulder, and low-back pains among medical students at Jazan University, Saudi Arabia: a cross-sectional study. J family Med Prim care.

[40] Alshagga MA (2013). Prevalence and factors associated with neck, shoulder and low back pains among medical students in a Malaysian Medical College. BMC Res Notes.

[41] Smith DR (2005). Musculoskeletal disorders among chinese medical students. Kurume Med J.

[42] Vujcic I et al. *Low back pain among medical students in Belgrade (Serbia): a cross-sectional study* Pain Research and Management, 2018. 2018. 10.1155/2018/8317906 PMC5829428 29623146

[43] Falavigna A (2011). Increased prevalence of low back pain among physiotherapy students compared to medical students. Eur Spine J.

[44] Hafeez K (2013). Back pain–are health care undergraduates at risk?. Iran J public health.

[45] Smuck M (2014). Does physical activity influence the relationship between low back pain and obesity?. Spine J.

[46] Yang H, Haldeman S (2018). Behavior-related factors associated with low back pain in the US adult population. Spine.

[47] Noormohammadpour P (2017). Prevalence of chronic neck pain, low back pain, and knee pain and their related factors in community-dwelling adults in Iran. Clin J Pain.

[48] Bath B (2014). Demographic and health characteristics of rural-and urban-dwelling canadians with chronic back disorders: a population-based comparison. Spine.

[49] Palacios-Ceña D (2015). Prevalence of neck and low back pain in community-dwelling adults in Spain: an updated population-based national study (2009/10–2011/12). Eur Spine J.

[50] Skillgate E (2017). Healthy lifestyle behavior and risk of long duration troublesome neck pain or low back pain among men and women: results from the Stockholm public health cohort. Clin Epidemiol.

[51] Bohman T (2014). Does a healthy lifestyle behaviour influence the prognosis of low back pain among men and women in a general population? A population-based cohort study. BMJ open.

[52] Tagliaferri SD (2020). Domains of chronic low back Pain and assessing treatment effectiveness: a clinical perspective. Pain Pract.

[53] Hoy D (2010). The epidemiology of low back pain. Best Pract Res Clin Rheumatol.

[54] Brown RCH, Maslen H, Savulescu J (2018). Responsibility, prudence and health promotion. J Public Health.

[55] Singhal K (2021). Do patients of chronic low back Pain have psychological comorbidities?. Avicenna J Med.

[56] Alsaadi SM (2014). The bidirectional relationship between pain intensity and sleep disturbance/quality in patients with low back pain. Clin J Pain.

[57] Sidiq M (2021). Prevalence of non-specific chronic low-back pain and risk factors among male soldiers in Saudi Arabia. PeerJ.

[58] Sribastav SS (2017). Interplay among pain intensity, sleep disturbance and emotion in patients with non-specific low back pain. PeerJ.

[59] Passos MH (2017). Reliability and validity of the brazilian version of the Pittsburgh Sleep Quality Index in adolescents☆. Jornal de pediatria.

[60] Longo UG (2021). Minimal clinically important difference and patient acceptable symptom state for the Pittsburgh Sleep Quality Index in Patients who underwent Rotator Cuff tear repair. Int J Environ Res Public Health.

[61] Kovacs F (2018). The association between sleep quality, low back pain and disability: a prospective study in routine practice. Eur J Pain.

[62] Akrouf Q et al. *Musculoskeletal disorders among bank office workers in Kuwait* EMHJ-Eastern Mediterranean Health Journal, 16 (1), 94–100, 2010, 2010. 20214165

[63] Hanna F (2019). The relationship between sedentary behavior, back pain, and psychosocial correlates among university employees. Front public health.

[64] Foley B (2016). Sedentary behavior and musculoskeletal discomfort are reduced when office workers trial an activity-based work environment. J Occup Environ Med.

[65] Aartun E (2016). The most physically active danish adolescents are at increased risk for developing spinal pain: a two-year prospective cohort study. BMJ open sport & exercise medicine.

[66] Bento TPF (2020). Low back pain in adolescents and association with sociodemographic factors, electronic devices, physical activity and mental health. Jornal de Pediatria.

[67] Dianat I, Alipour A, Jafarabadi MA (2017). Prevalence and risk factors of low back pain among school age children in Iran. Health promotion perspectives.

[68] Heneweer H, Vanhees L, Picavet HSJ (2009). Physical activity and low back pain: a U-shaped relation?. Pain.

[69] Verbunt JA, Huijnen IP, Köke A (2009). Assessment of physical activity in daily life in patients with musculoskeletal pain. Eur J Pain.

[70] Castro O (2020). How sedentary are university students? A systematic review and meta-analysis. Prev Sci.

[71] Amorim AB (2017). Does sedentary behavior increase the risk of low back pain? A population-based co-twin study of spanish twins. Spine J.

[72] Schwertner DS (2020). Prevalence of low back pain in young brazilians and associated factors: sex, physical activity, sedentary behavior, sleep and body mass index. J Back Musculoskelet Rehabil.

[73] Hanna F et al. *The Relationship between Sedentary Behavior, Back Pain, and Psychosocial correlates among University Employees*. Front Public Health, 2019. 7. 10.3389/fpubh.2019.00080 PMC6465323 31024881

[74] Brakenridge CL (2018). Evaluating short-term Musculoskeletal Pain changes in desk-based workers receiving a workplace sitting-reduction intervention. Int J Environ Res Public Health.

[75] Barone Gibbs B (2018). Reducing sedentary behaviour to decrease chronic low back pain: the stand back randomised trial. Occup Environ Med.

